# Microcystin Incidence in the Drinking Water of Mozambique: Challenges for Public Health Protection

**DOI:** 10.3390/toxins12060368

**Published:** 2020-06-02

**Authors:** Isidro José Tamele, Vitor Vasconcelos

**Affiliations:** 1 CIIMAR/CIMAR—Interdisciplinary Center of Marine and Environmental Research, University of Porto, Terminal de Cruzeiros do Porto, Avenida General Norton de Matos, 4450-238 Matosinhos, Portugal; isitamele@gmail.com; 2 Institute of Biomedical Science Abel Salazar, University of Porto, R. Jorge de Viterbo Ferreira 228, 4050-313 Porto, Portugal; 3 Department of Chemistry, Faculty of Sciences, Eduardo Mondlane University, Av. Julius Nyerere, n 3453, Campus Principal, Maputo 257, Mozambique; 4 Faculty of Science, University of Porto, Rua do Campo Alegre, 4069-007 Porto, Portugal

**Keywords:** drinking water quality, microcystin, Mozambique, public health

## Abstract

Microcystins (MCs) are cyanotoxins produced mainly by freshwater cyanobacteria, which constitute a threat to public health due to their negative effects on humans, such as gastroenteritis and related diseases, including death. In Mozambique, where only 50% of the people have access to safe drinking water, this hepatotoxin is not monitored, and consequently, the population may be exposed to MCs. The few studies done in Maputo and Gaza provinces indicated the occurrence of MC-LR, -YR, and -RR at a concentration ranging from 6.83 to 7.78 µg·L^−1^, which are very high, around 7 times above than the maximum limit (1 µg·L^−1^) recommended by WHO. The potential MCs-producing in the studied sites are mainly *Microcystis* species. These data from Mozambique and from surrounding countries (South Africa, Lesotho, Botswana, Malawi, Zambia, and Tanzania) evidence the need to implement an operational monitoring program of MCs in order to reduce or avoid the possible cases of intoxications since the drinking water quality control tests recommended by the Ministry of Health do not include an MC test. To date, no data of water poisoning episodes recorded were associated with MCs presence in the water. However, this might be underestimated due to a lack of monitoring facilities and/or a lack of public health staff trained for recognizing symptoms of MCs intoxication since the presence of high MCs concentration was reported in Maputo and Gaza provinces.

## 1. Introduction

**Figure 1:**
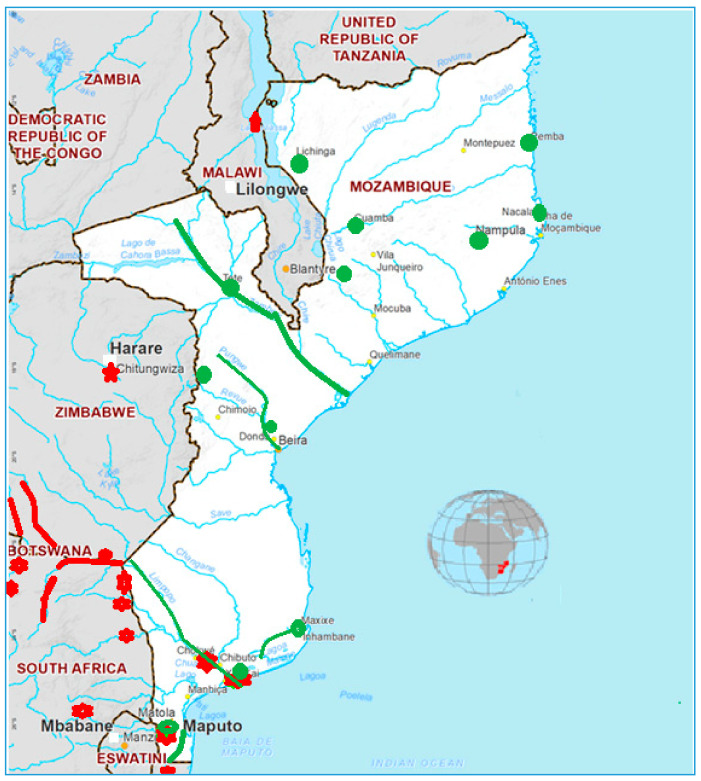
Map of Mozambique. Red points or lines indicate the sites where Microcystin (MC) or MC producers were detected in Mozambique, and in near sites or in the shared rivers with Mozambique, Green points indicate the water sources or water treatment centers.

Mozambique (Figure 1) is a country located in southeastern Africa (10°30′–26°52′ S and 40°50′–30°31′ E) covering a total land area of 800,000 km^2^. It is bathed by the Indian Ocean in the east and makes borders with Tanzania in the north; Malawi and Zambia in the northwest; Zimbabwe in the west and Swaziland and South Africa in the southwest. 

According to IV populational census carried out in 2017, this country has 29.67 million habitants distributed in 11 provinces [1]. The climate of Mozambique varies from subtropical climates (north and center) to dry arid (south) [2]. Like many African countries, Mozambique is highly vulnerable to climate variability and extreme weather events (droughts, floods, and tropical cyclones) [3]. Droughts are the most frequent natural disaster that have a negative impact on the population that reside in these rural areas [4]. The location of Mozambique in the coastal area makes it vulnerable to floods since many transnational river basins end [2]. Unfortunately, only 50% of the population has access to “safe drinking water”. Urban areas are the most favored, with 80%, while rural and most of the population have only 35% coverage and consume untreated water daily from rivers, lakes, and small puddles that form after or during the rain [5,6,7], putting at risk public health.

Eutrophication of freshwater resources may lead to the occurrence of cyanobacterial blooms and the presence of cyanotoxins, being microcystins (MC), the most common toxins worldwide [8]. The presence of MC in untreated drinking water is a major threat to public health because this potent cyanotoxin causes hepatotoxicity in humans. Thus, this review evaluates the incidence of MC and its producers in drinking water bodies of Mozambique, based on reported and available data and the estimated human illness case numbers and associated economic damage caused by Microcystin both overall globally, and in Mozambique—Africa. Recommendations for routine control and monitoring of MC will also be done since this hepatotoxin is not included in water control tests data in Mozambique.

## 2. Microcystin-Producing Species and Toxicology

### 2.1. Microcystin-Producing Species 

MCs are secondary metabolites produced by cyanobacteria species that occur naturally (but it can be increased severely by human activities) in freshwater environments. The most reported cyanobacteria species, which produce MCs are listed in Table 1 and include species of the families *Microcystaceae, Nostocaceae, Microcoleaceae, Oscillatoriaceae, Pseudanabaenaceae* (Table 1). The occurrence and development of a particular genus and species of cyanobacteria and cyanotoxins production worldwide seem to be conditioned to water chemistry and climate conditions [8]. In a temperate climate, *Microcystis* and *Anabaena* blooms occur widely while *Cylindrospermopsis* develops in tropical regions [9]. There are toxic and non-toxic cyanobacteria of the same species, which may be found together [8,10,11]. Toxic cyanobacteria can produce several toxins with different toxicity making it uncertain to assess the overall toxicity of bloom due to the variations of toxins concentration spatially and seasonally [12]. To distinguish toxic and non-toxic cyanobacteria species is very complicated, and consequently, the methods used are also complex. It implicates that the prevention of cyanobacteria bloom development is a suitable way to control toxic blooms [13,14].

The factors that promote the MC synthesis are not yet clearly understood, however, the optimal growth of MC-producing species and toxicity seem influenced by light intensity, nutrients, and temperature, among other factors. For example, the higher toxicity of *M. aeruginosa* extracts was verified in extreme pH values [39,40], and heavy metals such as Zinc and Iron did not influence the *M. aeruginosa* toxicity [41]. The content of nitrogen and phosphorus influenced the toxicity of *M. aeruginosa* extracts. Low nitrogen content reduces the *M. aeruginosa* toxicity, while low phosphorous increased the toxicity in the natural population [42,43] and reduced in lab experiments [16,21,44,45]. Another lab conclusion was the correlation of colony size and content of toxic cyclic heptapeptide of the non-axenic strain of *M. viridis* and axenic *M. viridis* was also verified [20,46,47]. In general, the optimal temperature for which MC-producing species produce MC ranged from 20 to 25 °C [21,40,48,49]. This range of optimal temperature suggests that cyanobacteria blooms are most toxic during periods with warm weather and in areas with warm climates [8]. 

### 2.2. Toxicology

**Figure 2:**
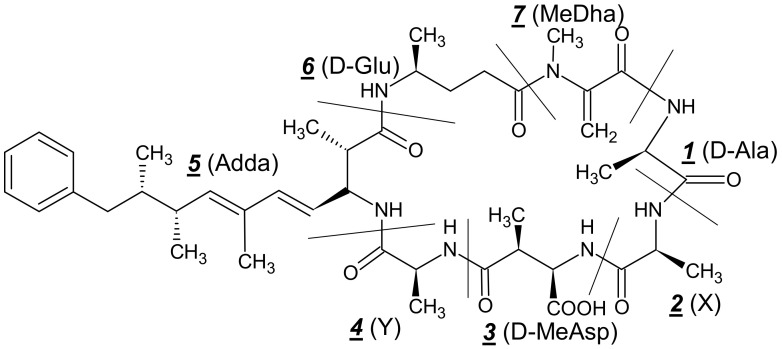
General chemical structure of microcystins. The common MC variant is MC-LR when X and Y correspond to L-Leu and L-Arg.

Microcystins (Figure 2) are the largest diverse group of cyanobacterial toxins, and to date, more than 240 MCs analogs are known, and they vary structurally in terms of the degree of methylation, hydroxylation, epimerization, peptide sequence, and consequently in their toxic effects [50,51,52]. Chemically, MC is a group of monocyclic heptapeptides (numbered in Figure 2) containing both D- and L-amino acids plus N-methyldehydroalanine (Mdha) and a unique β-amino acid side-group, 3-amino-9-methoxy-2-6,8-trymethyl-10-phenyldeca-4,6-dienoic acid (Adda) and their analogs differ among them, at the two L-amino acids and on the methyl groups on D-erythro-β-methylaspartic acid (D-MeAsp) and Mdha with molecular weight varying from 900 to 1100 Daltons. MC-LR, MC-RR, and MC-YR are common MC variants, the letters L, R, and Y represent the aminoacids leucine, arginine, and tyrosine, which appear on the MC molecule in different combinations [50,53,54,55,56,57,58] being MC-LR the most studied. The biosynthesis of this group of cyanotoxin is regulated by non-ribosomal peptide synthetase and polyketide synthase domains, being *MCyS* the gene cluster, which has been sequenced and partially characterized in several cyanobacterial species of the family *Microcystaceae, Nostocaceae, Microcoleaceae, Oscillatoriaceae, Merismopediacea,* and *Pseudanabaenacea* [16,17,18,19,20,21,22,23,24,25,26,27,28,29,30,31,32,33,34,35,36,37,38,59,60]

The mechanism of MCs toxicity seems to be well understood. They bind to serine/threonine-specific protein phosphatases (PPs) such as PP1 and PP2A, inhibiting their activity [61,62,63]. Adda moiety (Figure 1) plays an important role in the MC toxicity group since its isomerization and/or oxidation reduces the toxicity [64,65]. The inhibition of PP1and PP2A as a result of MC acute exposure causes excessive protein phosphorylation, alterations in the cytoskeleton, loss of cell shape, and consequently destruction of liver cells leading to intrahepatic hemorrhage or hepatic insufficiency [58]. Oxidative stress increasing in cells and consequent apoptosis, which can cause tumor promotion, is another mechanism of MC toxicity [66,67,68].

## 3. Effects of Microcystin in Humans, Symptoms, and Treatment

Microcystin effects in humans depend on the time of exposure and concentration ingested [69], and the studies are based on epidemiologic data but the reported studies on laboratory animals. Human health problems are mostly caused by chronic exposure by consumption of contaminated water or food, dermal exposure, or inhalation [57]. MC human poisoning episodes were reported in different parts of the world after the consumption of contaminated water or during sport or recreational activities [59,70,71,72]. Some examples of MC human poisoning cases are described; America—in 1996, an episode of human intoxications by MC was reported in Brazil with more than 76 deaths of patients at two dialysis centers in Caruaru. The municipal water supplied to the dialysis centers was the source of MC [71,73,74,75]. In Argentina, a human poisoning caused by MC involving a young man after immersion in an intense bloom *Microcystis* sp lake during sport and recreational activities were recorded. Four hours after exposure, the patient showed nausea, abdominal pain, and fever, and 48.6 µg·L^−1^ of microcystin-LR was detected in the water samples [76]. Other cases were recorded in Uruguay (January 2015) involving a 20-month-old child and her family during recreational activities. These victims were admitted to the hospital with diarrhea, vomiting, fatigue, and jaundice and the analysis confirmed the presence of MC-LR (2.4 ng·g^−1^ tissue) and [D-Leu1]MC-LR (75.4 ng·g^−1^ tissue) explanted liver [77]. Africa—toxic cyanobacteria suspected intoxication cases were reported in Zimbabwe involving children that were hospitalized in the Hospital of Harare with gastroenteritis symptoms [78]. In Europe—121 people presented abdominal pain, nausea, vomiting, diarrhea, fever, headaches, and muscle pain after consumption of untreated water from the River Kavlingean in Sweden. In this case a bloom of MC—producing such as *Planktothrix agardhii* and *Microcystis* spp. was observed in the river [79]. The most affected human organ is the liver [57]. However, in vivo and in vitro studies indicated that the kidney and colon are also affected [80,81,82,83,84,85]. The symptoms generally reported in humans due to the MC intoxication include gastroenteritis and related diseases, allergic and irritation reactions, liver diseases, tumors, and primary liver cancer and colorectal cancers, and massive hepatic hemorrhage. MC human poisoning treatment is very complicated due to the rapid, irreversible, and severe liver damage [86], however, gastric lavage [87], administration of monoclonal antibodies against MC-LR [88], immunosupressant Cyclosporine A, antibiotic rifampin [89], and membrane-active antioxidant vitamin E, taken as a dietary supplement [90] are recommended.

## 4. Microcystin Detection and Monitoring in Freshwater

According to the World Health Organization (WHO) guideline, the permitted limit of MC-LR for drinking water is 1.0 µg·L^−1^, and the tolerable daily intake is 0.04 µgKg^−1^ [91]. There are several MC detection methods, the most reported are listed in Table 2. Immunoassays (IA) are suitable methods of MC detection in Mozambique because they do not require sophisticated laboratory equipment and have a limit of detection below the maximum limit (1 µg·L^−1^). Additionally, IA can be used in both laboratory and field studies.

## 5. The Occurrence of Microcystin in Mozambican Drinking Water

### 5.1. The Drinking Water Scenario in Mozambique

The drinking water supply scenario in Mozambique still faces major challenges because a majority of the population still consumes untreated drinking water and consequently is exposed to many water-borne diseases. Only 50% of the population has access to “safe drinking water”. Urban areas are the most favored, with 80%, while rural and most of the population have only 35% coverage and consume untreated water daily from rivers, lakes, and small puddles that form after or during the raining season [5,7]. The low water supply cover in Mozambique is inconceivable due to several reasons, among them, the existence of natural water cover (rivers) in the whole country and the presence of excessive fragmentation of governmental organisms for water management (Figure 1). The water management is led by the Ministry of Public Works, Habitation and Hydric Resources (MOPHRH), which operates, among others, with the National Direction of Water Supply and Sanitation, National Direction of Hydric Resource Management, Water Regional Administrations, Sanitation, and Water Supply Infra-structure Administration, Water Regulation Council, Fund for Investment and Patrimony of Water Supply, and other private institutions, which provide goods and services. In order to improve the water management and expand the coverage, different projects funded by the Mozambican government and non-governmental organizations such as Plataforma Moçambicana de Água [6], Greater Maputo Water Supply Expansion Project [110], Integrated Water Supply and Sanitation Project for the provinces of Niassa and Nampula [111], National Rural Water Supply and Sanitation Program (PRONASAR) in Nampula and Zambezia Provinces [112], Inhambane Rural Water Supply and Sanitation Program [113], and others were implemented involving all the MOPHRH, civil society, and private organisms. However, still to date, the national water supply does not cover enough, with the population still consuming untreated water.

The water policy in Mozambique was approved in 1995, revised in 2007 and 2016, which, in the scope of water supply and sanitation, has the following relevant goals [114]:

- Achieve the sustainable development goals, universal access to water supply, and sanitation.
- Meeting of the basic needs of the poorest population, to reduce poverty, always looking for a sustainability situation.
- Water valuing, not only as a social and environmental asset but also with the economic value it holds.
- Government’s concentration on the definition of priorities, standards, regulation, and promotion of the private sector.
- Development of an institutional framework that contributes to the management of water as a resource and provision of decentralized and autonomous water supply and sanitation, where the private sector is called upon to participate.

The Ministry of Health (MH) is the legal organism responsible for water quality control and follows the regulation of the WHO, which sets the parameters of the quality of water intended for human consumption and the methods of carrying out their checks in order to protect human health. The water quality control recommended by WHO include MC among other biological parameter and the provisional guideline value is 1 µg·L^−1^ for drinking water [115]. The challenge is enormous in Mozambique for control or monitoring of this hepatotoxin due to the lack of adequate laboratories for the detection of MC in drinking water, even for 50% of the population that consumes treated water. This scenario shows clearly that all the Mozambican population is very vulnerable to MC exposure.

### 5.2. Microcystin in Mozambican Drinking Water

The drinking water is supplied by private (autonomous systems) and governmental operators. In Table 3, are listed the main sources of drinking water in Mozambique and includes underground and water river. The drinking water treatment is performed mainly by disinfection with chlorine, but is some regions such as Pemba and Niassa, the water treatment system includes the removal of iron by aeration. Not only is there no drinking water treatment for MC removal, but also MC incidence data in Mozambique are very limited. However, according to the WHO, more than 500,000 cases of diarrhea were reported, which 100 and 7 cases correspond to dysentery and cholera, respectively, and others are unknown [116].

These data indicate that many people of Mozambique consume food and water unsafely. Few studies (Figure 1) were done by Pedro et al. [117,118,119] and Bojcevska and Jergil [120] in Pequenos Libombos dam, Nhambavale lake, Chòkwé irrigation channels and Chidenguele sites in the South of Mozambique during 2003, 2008, and 2009 and their studies indicated the occurrence of MC-LR, -YR, and -RR produced by *Microcystis* sp. (*M. novacekii, botrys* and other) and *Cylindrospermopsis raciborskii* [117,118,119,120] (Table 4). MC concentration varies from less than 0.01 (below quantification levels) to 0.02 in Pequenos Libombos dam, less to 0.01 to 0.68 in Chòkwé irrigation channels, 0.86 to 7.82 in Nhambavale lake and 0.57 to 6.83 µg·L^−1^ in Chidenguele. Higher MC concentration values than the maximum limit ranging from 6.83 to 7.78 µg·L^−1^ (around 7 times above) were found in the Nhambavale lake and Chidenguele sites. These data highlight (suggest) the need to implement an operational monitoring program of MCs since the tests recommended by MH do not include the MC test [121]. Neighboring countries published other data, which support the need for MC monitoring in Mozambique (Figure 1), namely:

- **South Africa**: MC Producers: *Synechocystis* sp. *Microcystis aeruginosa, Microcystis panniformis, Nostoc* sp., *Planktothrix* sp., *Phormidium* sp., in the Limpopo river basin [122,123,124,125,126], Hartbeespoort dam [127,128,129,130,131], Kruger National Park [132], Sand, Mawoni, Lephalale, Mokolo, Crocodile, Nzhelele ivers [126] MC -YR, -LR, -FR, -YA, -LA, -LAba (0.156–0.270, 0.059–0.18, 0.09, 0.02–0.044, 0.051–0.241, 0.080 mg.g^−1^) in Natal and Transvaal dams [133], 8.6 µg·L^−1^ in Hartbeespoort dam [134], 12,300 µg·L^−1^ in Hartbeespoort lake [135].
- **Tanzania**: MC-LR and -RR in different tissues of dead flamingos (*Phoeniconaias minor*) from Empakai Crater, Lake Natron and Lake Manyara (0.165–1.16 ng.g^−1^) [136,137,138], MC-RR (0.4–13 µgL^−1^) in Victoria lake [139,140], MC producers: *Aphanocapsa* sp., *Anabaena* sp., *Microcystis* sp. in Victoria lake [139,140].
- **Zimbabwe**: MC producers: *Microcystis aeruginosa* in Mzingwane river, Shashe River [126], *Microcystis* *wesenbergii* [141,142]. MC-LR (1.62–22 µgL^−1^) in Chivero lake [141,142].
- **Malawi**: MC producers: *Anabaena* sp. in Malawi lake [143].

For example, the drinking water in the Xai Xai district (Gaza province) (Figure 1) is supplied from the Limpopo river. This river contains different MC producers such as *Synechocystis* sp. *Microcystis aeruginosa*, *Microcystis panniformis*, *Nostoc* sp., *Planktothrix* sp., *Phormidium* sp., which were detected in South Africa areas [122,123,124,125,126]. The presence of a potentially toxic algae is not an indication of MC production but is an indication of the need for MC screening in order to confirm the MC presence.

### 5.3. Removal of Microscystin from Drinking Water in Mozambique

MCs can be removed from drinking water using several rapid and low-cost. The most are reported in laboratory studies, and they are not adaptable to economic conditions in Mozambique [144,145,146,147,148,149,150]. However, the following techniques seem to be useful in Mozambique and can be implemented in both rural and urban zones: Photolysis at 254 and 185 nm [147], use of wood-based and coconut-based activated carbons [148], use of bamboo-based charcoal adsorbent modified with chitosan [149], hydrophyte filter bed [150], biological activated carbon process [151], aquatic vegetable bed [152], and activated carbon from the seed husks of the pan-tropical tree, *Moringa oleifera* [153], among others.

## 6. Final Considerations and Recommendations 

The drinking water supply scenario in Mozambique still faces major challenges because the majority of the population still consumes untreated drink water (from rivers, lakes, and small puddles that form after or during the raining season) and consequently exposed to many water diseases [5,7] including, for example, gastroenteritis, which is caused by hepatotoxins MCs. To date, no data of water poisoning episodes recorded were associated with MCs presence in the water. However, this might be underestimated due to a lack of monitoring facilities and/or a lack of public health staff trained for recognizing symptoms of MCs intoxication since the presence of high MCs concentration was reported in Maputo and Gaza provinces. Few studies done in Maputo and Gaza provinces indicated the occurrence of MC-LR, -YR, and -RR at a concentration ranging from 6.83 to 7.78 µg·L^−1^ [117,118,119,120], which are very high, around 7 times above the maximum limit (1 µg·L^−1^) recommended by WHO [59]. The potential MC-producing in the studied sites is mostly *Microcystis* sp. [117,118,119,120]. However, MC distribution in Mozambique is unknown, and a monitoring program would help to understand the dimension of the problem. To date, no water MC poisoning episodes data recorded in Mozambique. The absence of MC intoxication episodes might be underestimated due to the absence of MC monitoring plan and/or a lack of public health staff trained in recognizing symptoms of MC intoxication. MC monitoring may be implemented according to recommendations of WHO [59] (1 µg·L^−1^), and the respective MC analysis can be done in the existing water treatment centers in each province (Table 3). Rapid tests for MC detection, such as ELISA, can be used in each center. In the case of higher MCs content, some suitable techniques for MC removal may be used. The recommended techniques include photolysis at 254 and 185 [147], the use of wood-based and coconut-based activated carbons [148], use of bamboo-based charcoal adsorbent modified with chitosan [149], hydrophyte Filter Bed [150], Biological Activated Carbon Process [151], and aquatic vegetable bed [152], among others. These techniques can be used in both rural and urban areas due to their low-cost implementation and local access.

## Figures and Tables

**Table 1:** Microcystin-producing species detected in freshwater bodies.

Order	Family	Species
*Chroococcales*	*Microcystaceae*	*Microcystis* sp. [15], M. *aeruginosa* [16,17,18,19,20,21,22,23,24,25,26], *M. viridis* [20,24], *M. wesenbergii* [25,27], *M.* spp. [28,29], *M. ichthyoblabe* [25] and *Synechocystis* sp. [25]
*Nostocales*	*Nostocaceae*	*Anabaena* spp. [29,30], *A. flos-aquae* [23,27], *A.* sp. [15,31], *A. subcylindrica* [32], *A, variables* [32], *Nostoc* sp. [27], *Aphanizomenon flos-aquae* [23,29,33] and *A. circinalis* [34]
*Oscillatoriales*	*Microcoleaceae*	*Planktothrix prolifica* [24] and *P. agardhii* [29]
	*Oscillatoriaceae*	*Oscillatoria agardhii* [35], *O. limosa* [36], *O. chlorina* [25], *Phormidium konstantinosum* (*O. tenuis*) [36], *P. corium* [32] and *Plectonema boryanum* [32]
*Synechococcales*	*Merismopediaceae*	*Synechocystis aquatilisf. salina* [37] and *Aphanocapsa cumulus* [38]
	*Pseudanabaenaceae*	*Pseudanabaena mucicola* [25] and *P. galeata* [25]

**Table 2:** MC detection methods in drinking water. IA—immunoassays, HPLC—high-performance liquid chromatographic, PAD—photodiode-array detector, LC—liquid chromatography, MS—mass spectroscopy, MALDI-TOF MS—matrix-assisted laser desorption/ionization time-of-flight mass spectrometry, UV—ultraviolet detector.

MC Variant	Detection	LOD	LOQ	Reference
-LR: -LY: -LW: -LF: -LA: Asp^3^(Z)-Dhb^7^-HtyR: -DAsp^3^-RR	IA	50–20,000 pg·mL^−1^		[88,92,93,94,95,96,97,98,99,100,101,102]
-RR: -LR: -LY: -LF	HPLC-UV			[102]
-RR: -LR: -LY: -LW: -LF: -FR; -WR	HPLC-PAD	5 ng		[103,104]
3-demethyl-MC-LR: -LR: -LY: -LA: -LW: -LF: 3-demethyl-MC-RR: -RR: 3-demethyl-MC-YR: -YR	LC–MS (/MS)	0.2 pg–2057 pg	1pg–15 µg·L^−1^	[93,102,104,105,106,107,108]
D-MC-LR; -LR: D-MC-RR: D-MC-YR: -RR: -YR: [H4]MC-YR: -WR	MALDI-TOF MS			[109]

**Table 3:** Treatment and drinking water supply in Mozambique. Gov—Government system. HTH—High test hypochlorite [7,114].

Province	Water System	Water Treatment Center	Capacity, m^3^·dia^−1^	Water Source	Supplied Sites
Maputo	Gov-Umbeluzi	Umbeluzi	240,000	Umbeluzi river and Pequenos Limbobo Dam	Maputo, Matola and Boane
	Ka Tembe Autonomous	Ka Tembe	760	Underground – Ka Tembe	Ka Tembe
	Vila Olimpia Autonomous		-	Underground - Maputo	Vila Olimpia
	The Small		6500	Underground -Maputo	Zona Verde, Kongolote, Matola Gare na Matola, Magoanine and Albazine
Gaza	Gov-Xai-Xai	Xai-Xai	22,790	Limpopo river	Bairro 11, Bairro 13, Hospital, Patrice Lumumba, Inhamissa 6, CFPP, Marieny Gouaby, Chinuguine and Praia
	Gov-Limpopo				
	Gov-Chongoene				
	Xai-Xai Autonomous			Underground – Xai-Xai	Chicumbane, Julius Nyerere, Muahetane e Chongoene
	Gov-Chókwè		10,056	Limpopo river and underground - Chokwe	Lionde, Conhane, Massavassa, Nwachicoluane, Xilembene, Hókwe, Mapapa
	Chókwè Autonomous		6816		
	Gov-Guija			Underground - Guija	vila-sede do distrito de Guijá
Inhambane	Gov-Inhambane		11,176		Inhambane City, Salela, Nhamua e Josina Machel
	Gov-Maxixi		9120	Inhanombe river	Chambone, Rumbana, Nhambiho, Bato, Habana, Malalane, Macupula, Macuamene, Maquetela, Eduardo Mandlane, Nhamaxaxa, Matadouro, Mabil, Barrane and Bembe
	Mangapana and Mabil Autonomous				Mangapana and Mabil
Sofala	Beira and Dondo	Mutua	50,000	Pungué river	Beira and Dondo
Manica	Gov-Manica	Chicamba	38,600		Manica, Chimoio and Gondola and Messica and Bandula village
Tete	Gov-Tete	Tete: Aeration through a cascade, followed by two decantation tanks and then filtration and finally disinfection with granular chlorine	38,495	Zambeze river	Tete city
	The Degué small				Degué
Zambezia	Gov-Zambezia	Licuar: Disinfection with HTH	19,512	Underground - Licuar	Quelimane, Nicoadala and Licuar
Nampula	Gov-Nampula	Nampula: Pre-chlorination, flocculation, decanting and filtration	20,000	Monapo dam	Nampula city
	Gov-Nacala	Nacala: A mixture of flocculation, decantation, filtration, and disinfection	6000	Nacala dam	Nacala city
Cabo Delgado	Gov-Pemba	Pemba: Removal of iron by aeration and filtration	12,000	Underground-Metuge	Pemba city
	Gov-Angoche	Angoche: Disinfection with HTH	1800	Underground-Malatane	Angoche
Niassa	Gov-Lichinga	Locumué	2400	Locumué dam	Lichinga
	Chiuaula Autonomous			Underground - Chiuala	Chiuaula
	Cuamba	Cuamba: Disinfection with HTH	960	Mpopole dam	Cuamba

**Table 4:** The Incidence of Microcystin and its producers in the aquatic environments of Mozambique. PL—Pequenos Libombos dam, NL—Nhambavale lake, CH—Chòkwé irrigation channels, RFLP—restriction fragment length polymorphism, MC—microcystins, ELISA—enzyme-linked immunosorbent assay, CG—Chidengule, LM—light microscope, PCR— polymerase chain reaction, ML—Malawi lake, NL— Niassa lake.

Local	Date	Producer		MC			Reference
		Species	Detection	MC Variant	Detection	Conc.	
PL	2008–2009	*Microcystis* sp.	PC gene	LR and YR	LC-MS	3.9 ng·g^−1^	[118]
		*Microcystis* sp.	MCyA-MISY gene				
		*Microcystis* sp.	MCyB gene				
		*Microcystis* sp.	RFLP				
NL		*Microcystis* sp.	PC gene	LR, YR and RR		159.4 ng·g^−1^	
		*Microcystis* sp.	MCyA-MISY gene				
		*Microcystis* sp.	MCyB gene				
		*Microcystis* sp.	RFLP				
CH		*Microcystis* sp.	PC gene	LR		2.7 ng·g^−1^	
		negative	MCyA-MISY gene				
		negative	MCyB gene				
		negative	RFLP				
PL	2002		LM	MC	ELISA	0.22 µg·L^−1^	[120]
CH		*Cylindrospermopsis raciborskii*				< 0.01 µg·L^−1^	
CG		*Microcystis novacekii* and *M. botrys*				6.83 µg·L^−1^	
PL	2008–2009			LR	LC-MS	< 0.01 µg·L^−1^	[117]
				YR		0.01 µg·L^−1^	
CH				LR		0.68 µg·L^−1^	
				YR		0.06 µg·L^−1^	
NL				LR		7.78 µg·L^−1^	
				YR		0.07 µg·L^−1^	
				RR		< 0.01 µg·L^−1^	
PL	2008–2009	*Microcystis aeruginosa*	PC gene				[119]
			MCyB-Taq-Nuclease assay				
NL			PC gene				
			MCyB-Taq-Nuclease assay				
CH			PC gene				
			MCyB-Taq-Nuclease assay				
ML/NL	2002	*Anabaena* sp.	LM				[143]
NKP	2007	*Microcystis aeruginosa*	PCR	LR	ELISA	23718 μg·L^−1^	[132]
